# Sickle Cell Disease in Africa: SickleInAfrica Registry in Ghana, Nigeria and Tanzania

**DOI:** 10.1002/jha2.70044

**Published:** 2025-05-06

**Authors:** Jack Morrice, Wilson Mupfururirwa, Reuben I. Chianumba, Evans Xorse Amuzu, Daniel Kandonga, Victoria Nembaware, Mario Jonas, Jade Hotchkiss, Upendo Masamu, Arthemon Nguweneza, Bruno P. Mmbando, Irene Minja, Agnes Jonathan, Nicola Mulder, Emmanuel Balandya, Alex Osei‐Akoto, Vivian Paintsil, Julie Makani, Obiageli Nnodu, Members of SPARCO Nigeria, Raphael Z. Sangeda, Andre Pascal Kengne, Gaston Kuzamunu, Ambroise Wonkam

**Affiliations:** ^1^ Division of Human Genetics Department of Pathology University of Cape Town, Health Sciences Campus Observatory South Africa; ^2^ Centre of Excellence for Sickle Cell Disease Research & Training University of Abuja (CESRTA) Abuja Federal Capital Territory Nigeria; ^3^ Directorate of Child Health‐Komfo Anokye Teaching Hospital Kumasi Ghana; ^4^ Sickle Cell Programme Muhimbili University of Health and Allied Sciences Dar es Salaam Tanzania; ^5^ Department of Integrative Biomedical Sciences Computational Biology Division IDM, CIDRI‐Africa WT Centre, University of Cape Town, Health Sciences Campus Observatory South Africa; ^6^ Faculty of Health Sciences Institute of Infectious Disease & Molecular Medicine University of Cape Town Cape Town South Africa; ^7^ Department of Physiology Muhimbili University of Health and Allied Sciences Dar es Salaam Tanzania; ^8^ Child Health Directorate, Komfo Anokye Teaching Hospital Kumasi Ghana; ^9^ Department of Clinical Microbiology School of Medicine and Dentistry Kwame Nkrumah University of Science and Technology Kumasi Ghana; ^10^ Non‐communicable Diseases Research Unit South African Medical Research Council, Durban and Cape Town Cape Town South Africa; ^11^ African Institute for Mathematical Sciences Cape Town South Africa; ^12^ McKusick‐Nathans Institute and Department of Genetic Medicine Johns Hopkins University School of Medicine Baltimore Maryland USA

**Keywords:** disease management, meta‐analysis, registry, regression models, sickle cell disease, SickleInAfrica

## Abstract

**Introduction:**

Sickle cell disease (SCD) is most prevalent in Sub‐Saharan Africa (SSA), where incomplete patient profiles and limited management strategies hinder research and healthcare standards.

**Methods:**

We describe the first large‐scale and multinational assessment of 13,403 SCD patients enrolled from 2017–2021 across 31 facilities in Ghana, Nigeria, and Tanzania into the SickleInAfrica consortium registry. We used hierarchical regression models to estimate and analyze the demographics, adoption levels of SCD diagnosis and therapies.

**Results:**

The average age at diagnosis was 3 months, 19 months and 3 years in Ghana, Nigeria and Tanzania respectively, reflecting differences in country‐specific newborn screening programs and policies. Hydroxyurea (HU) use was highest in Ghana (21%), followed by Nigeria (12%) and Tanzania (6%), with significant variability across facilities. Sex differences in SCD management were observed, with males more likely to receive HU and blood transfusions. At the consortium level, HU initiation correlated with enrolment age rather than age at diagnosis, highlighting the need for earlier intervention.

**Conclusions:**

Our findings highlight the potential of the SickleInAfrica registry toward enhancing understanding of regional disparities in SCD care and potential gender inequalities, emphasizing the need for enabling policies toward strengthened SCD research and improved quality of life and care of patients in Africa.

## 1 Introduction

The Sickle Cell Disease Ontology (SCDO) defines sickle cell disease (SCD) as a pleiotropic inherited blood disorder characterized by sickle‐shaped red blood cells and anemia [1, 2]. SCD occurs when an individual inherits two copies of the sickle cell mutation (Glu7Val; substitution of glutamic acid by valine at position seven of the hemoglobin beta‐chain encoded by the *HBB* gene), resulting in most cases to homozygous HbSS genotype. Alternatively, SCD can arise from compound heterozygous genotypes, where one sickle gene (HbS) is inherited alongside a different hemoglobin variant mutation (e.g., HbSC) or a beta‐thalassemia mutation (e.g., HbS/βThal) [3]. Rare dominant forms of SCD, such as SCD A/Jamaica Plain [SCDO:1000164], occur when the sickle mutation coexists with another mutation on the same *HBB* allele [4]. SCD causes lifelong clinical and psychosocial challenges, reducing quality of life and increasing mortality. Complications, primarily caused by capillary obstruction and inefficient oxygen delivery due to deformed red blood cells, include pain, infection susceptibility, anemia, and organ dysfunction [5, 6].

Multinational disease registries are essential for assessing SCD genotypes, complications, diagnostic age distributions, and adherence to management guidelines at various levels, guiding research, funding, and policy. While a few global SCD registries exist, Africa has lagged behind [7, 8]. The establishment of the SickleInAfrica registry is a major step in addressing knowledge gaps in sub‐Saharan Africa (SSA) [9, 10]. The SickleInAfrica consortium includes the Sickle Pan‐African Research Consortium (SPARCo), SPARCo Clinical Coordinating Centre (CCC), SickleInAfrica Data Coordinating Centre (SADaCC), and the SCDO working group [11]. SPARCo initially operated in Ghana, Nigeria, and Tanzania during Phase 1 (2017–2021) and expanded to include Uganda, Mali, and Zambia/Zimbabwe in Phase 2 (2021–2026). Supported by SPARCo CCC, SADaCC oversees a centralized sickle hemoglobinopathy registry and capacity building in data management, bioinformatics, and biostatistics. This study analyzed patients’ profiles and levels of SCD clinical care from Phase 1, examining variations in SCD management across the consortium by mapping diagnostic age distributions and the prevalence of basic therapies like hydroxyurea (HU). Regression models were employed to identify potential determinants, such as patient demographic variables, driving late diagnoses and low therapy adoption rates at country and consortium levels.

## 2 Methods

The Phase 1 consortium countries contributed patient record data from 31 facilities: Ghana (1 facility), Nigeria (22 facilities), and Tanzania (8 facilities). Phase 1 resulted in 1514 standardized data elements, with 92 of these being collected across all facilities (see Table S1). These data elements include collected patient SCD Hb genotypes, as well as the laboratory techniques used to determine the genotypes. In Ghana, isoelectric focusing (IEF) was used, while Tanzania used high‐performance liquid chromatography (HPLC) and hemoglobin electrophoresis (HbE), and Nigeria used IEF. After data from the three countries was cleaned, harmonized and filtered, only 14 out of 92 variables were available and complete across all facilities. These included six demographic variables (study identifier, country, facility, sex, age at enrolment into the registry, age at SCD diagnosis); five treatment/prophylaxis‐related variables (age of hydroxyurea (HU) therapy initiation, HU use, penicillin prophylaxis, malaria prophylaxis and folic acid use), and three hematology‐related variables (history of blood transfusion, SCD genotype, and blood group).

### 2.1 Study Populations and Sampling

For each of the 31 facilities in the study, we defined a unique population as the catchment population of that facility, restricted to those patients who are currently receiving care for SCD at that facility. Thus, the population of this registry study is composed of 31 distinct subpopulations, each with participants enrolled from public healthcare facilities. All SCD patients who were attending these clinics were eligible for enrolment.

### 2.2 Data Management and Sharing

Data was collected and managed using the Research Electronic Data Capture (REDCap) software at each SPARCo facility [8, 9]. At each facility, data cleaning and quality checks were carried out by the data entry and management personnel before being shared with SPARCo CCC and SADaCC. At SADaCC, data files were merged after harmonization using the SickleInAfrica standardized data elements (https://www.sickleinafrica.org/SIA_data_elements). Duplicate records were removed, different encodings for missing values were harmonized, free text responses were sorted into nonoverlapping categories, and dates were reformatted for consistency. All data cleaning, harmonization, and analysis were performed at SADaCC and SPARCo CCC using the R programming language (version 4.4.1).

### 2.3 Dataset Summary Statistics

The cleaned and harmonized registry data was analyzed hierarchically by geospatial location. Participants were first clustered by enrolment facility, then by country. Patient‐level variables were summarized for each enrolment facility as frequencies (percentages) for categorical variables, and median (with interquartile range [IQR]) for continuous variables. These summary values were then pooled together across enrolment facilities for each country by taking the median of percentages for categorical variables and the median of medians for continuous variables. These medians were then used to pool results across the countries. Distributions of the continuous variables were summarized with histograms, and an appropriate normalization procedure was chosen based on the observed distribution profiles.

### 2.4 Univariable and Multivariable Analyses

Parameter estimates for each variable were generated at the country level using hierarchical regression models with random intercepts, clustering by the enrolment facility catchment populations. Fixed‐effect estimates with 95% confidence intervals (CIs) and *p*‐values were pooled across imputed datasets using Rubin's rules, then combined across countries using meta‐analysis to produce consortium‐wide estimates. Inverse‐variance weighting with random effects accounted for heterogeneity across facilities and countries, which was assessed using Cochran's *Q* test and quantified with the *I* ^2^ statistic.

To test associations, hierarchical linear and logistic regression models were used. For SCD treatment regimens, hierarchical logistic regression models analyzed the impact of sex, with age at enrolment included as a covariate. A separate hierarchical linear regression model assessed the association between age at diagnosis and HU use (yes/no), restricted to HbSS participants and excluding those diagnosed in their first year of life to minimize bias from newborn screening (currently only available in Ghana). Participants from facilities with fewer than five individuals in any subgroup (e.g., males using HU) were excluded. Age at enrolment and sex were included as covariates in all models.

### 2.5 Survival Outliers

An important subclass of SCD patients is the treatment‐naive long survivors, or survival outliers. A survival outlier was defined as a participant with an abnormally late age‐at‐enrolment, diagnosis, and initiation of hydroxyurea treatment with respect to the participant's facility catchment population sample. Outliers for age‐at‐enrolment, age‐at‐diagnosis and age‐at‐hydroxyurea‐initiation were identified using the robust Mahalanobis distance method applied to a single imputed dataset at the facility level. This method uses the mean and covariance matrix of these target variables within each facility, where abnormality was determined relative to the mean. Participants exceeding the 97.5th percentile—approximately 2.24 standard deviations beyond the multivariate center—were classified as survival outliers. The Mahalanobis distance method accounts for correlations among these variables, ensuring that outliers are identified based on a multivariate distance rather than individual thresholds.

## 3 Results

### 3.1 Enrolment Facilities

**FIGURE 1:**
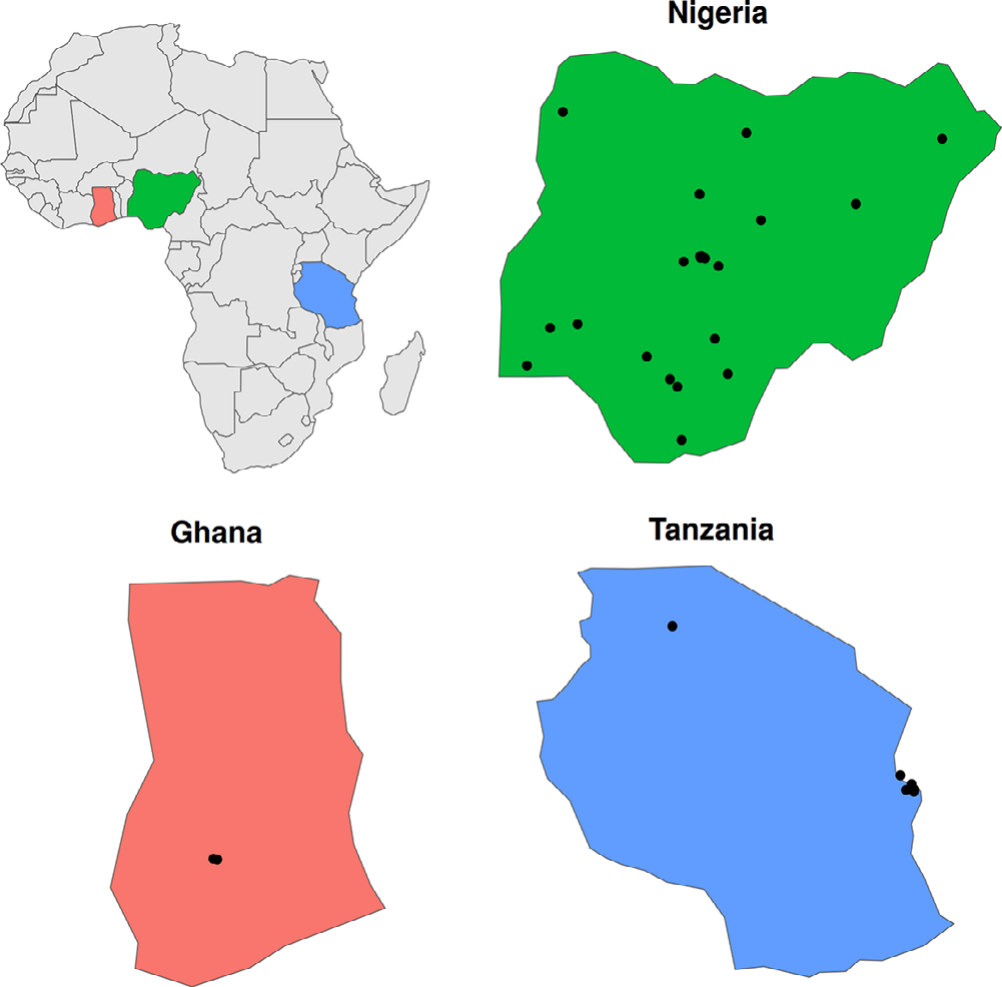
Spatial distribution of the registry enrolment facilities for the 3 consortium countries. Each black dot marks the location of an enrolment facility. Due to the high density of facilities in some regions, several black dots overlap. Specific location information of the facilities is available in Table S2.

Each SPARCo center first conducted a center‐specific registry analysis, and the results of these studies were published [8, 12, 13]. We present results from hierarchical regression analysis across all SPARCo centers (Figure 1 and Table S2). In Nigeria, the enrolment facilities cover all geopolitical zones. In Tanzania, facilities are clustered around Dar es Salaam, except for an outlying facility in Mwanza that serves patients from across the northwest regions of Tanzania [13]. The Ghana facility is located in Kumasi.

### 3.2 Demographic Characteristics

**FIGURE 2:**
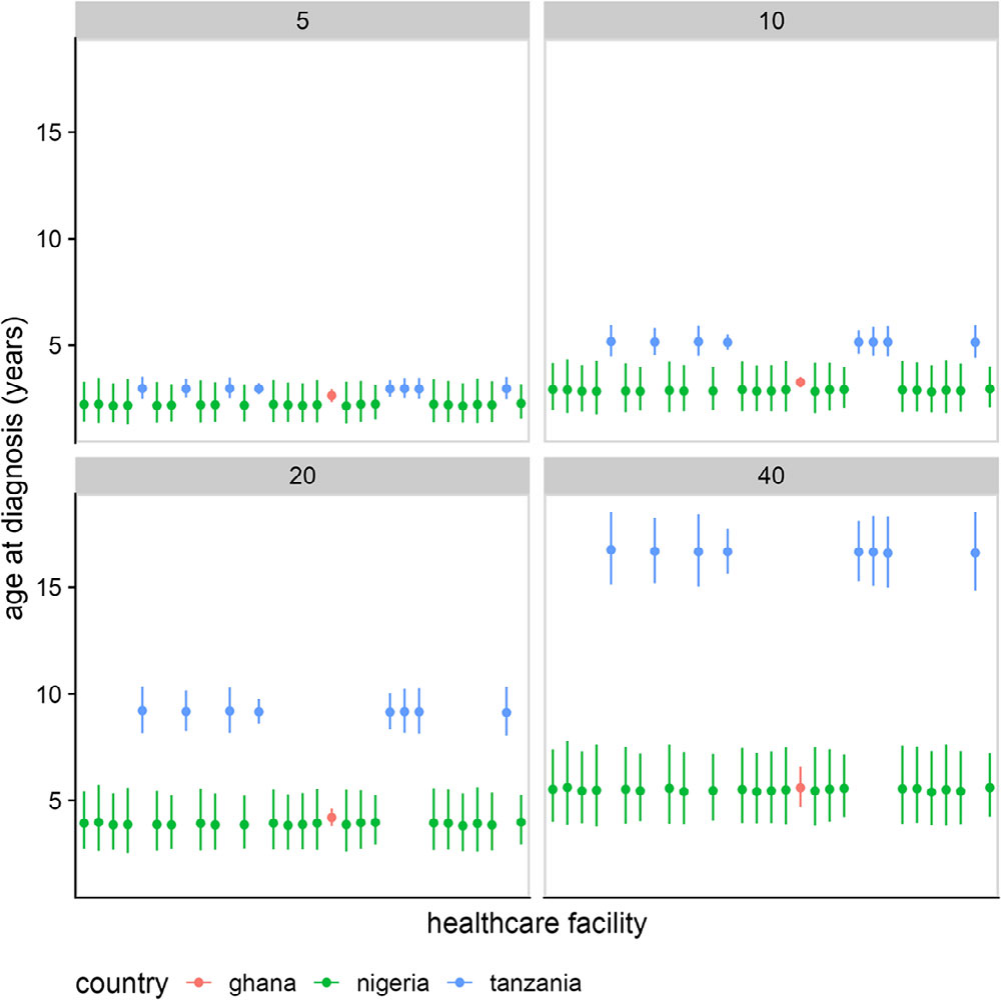
Predicted median age at diagnosis across healthcare facilities in the registry. This figure shows the median age at diagnosis for patients at different healthcare facilities, grouped by age at enrolment. Each panel represents a fixed age at enrolment: 5 years (top left), 10 years (top right), 20 years (bottom left), and 40 years (bottom right). Points indicate the median age at diagnosis for each facility, with bars representing 95% confidence intervals (CIs). Different colors represent different countries.

Enrolled participant profiles are summarized in Table 1. Distributions of age‐at‐enrolment differed significantly across the three countries (*I* ^2^ = 94.8, *p*‐value < 0.001). On average the youngest study populations were from Tanzania, with a country‐level median age of 8.50 (95% CI [7.46, 9.64]) years, and the oldest were from Nigeria with a median age of 13.63 years (95% CI [11.23, 16.36]). Significant heterogeneity was also observed between the distributions of age at SCD diagnosis in each country (*I* ^2^ = 98.9, *p*‐value < 0.001). On average, the earliest diagnoses occurred in Ghana, a country‐level median age of 0.25 (95% CI [0.17, 0.35]) years and the latest diagnoses occurred in Tanzania, a country‐level median age of 3.10 (95% CI [2.58, 3.68]) years. The oldest recorded age at diagnosis was 59, 29, and 57 years old in Nigeria, Tanzania, and Ghana, respectively. The (predicted) median age at diagnosis is consistently low in Nigeria and Ghana, and highest in Tanzania across all age groups, sexes, and healthcare facilities. For example, a participant with the HbSS genotype, enrolled in the study at 40 years old, is predicted to have been diagnosed at around 5 years of age if they were Nigerian or Ghanaian, but at almost 20 years if they were Tanzanian (see Figure 2).

**TABLE 1:** Univariable summary statistics for the SickleInAfrica registry. Summary statistics for each variable segregated by country, showing 95% confidence intervals (CIs) in brackets where relevant. Where possible, consortium‐wide estimates are shown in the “Overall” column. *I* ^2^ and *p*‐values from Cochran's Q test for heterogeneity are in the final two columns.

Variable	SPARCo Centre				Consortium *I* ^2^	Consortium heterogeneity *p*‐value
	Ghana	Nigeria	Tanzania	Overall		
Number of facilities	1	22	8	31		
Number of SCD patients enrolled	3143	6572	3683	13398		
Sex (males/females)	53% (51, 55)	48% (46, 51)	51% (49, 53)	51% (48, 54)	78.9	0.009

Age (in years), median						
at enrolment	9.17 (8.83, 9.52)	13.63 (11.23, 16.36)	8.50 (7.46, 9.64)	10.14 (7.57, 13.24)	94.8	< 0.001
at diagnosis	0.25 (0.17, 0.35)	1.60 (1.21, 2.07)	3.10 (2.58, 3.68)	1.28 (0.22, 3.81)	98.9	< 0.001
at hydroxyurea initiation	7.56 (7.00, 8.14)	11.42 (9.03, 14.19)	7.41 (6.44, 8.46)	8.48 (6.46, 10.89)	91.4	< 0.001

% Patients with SCD genotype						
HbSS	67 (65, 69)	99 (98, 99)	100 (96, 100)	97 (62, 100)	98.4	< 0.001
HbSC	33 (31, 34)	1 (0, 2)	< 0.01%	2 (0, 38)	99.1	< 0.001
HbS/βThal	< 0.01	< 0.01	< 0.01	< 0.01	—	—

% Patients using SCD treatment regimens						
Currently using hydroxyurea?	21 (19, 22)	8 (4, 15)	6 (2, 20)	12 (5, 24)	80.4	0.002
Blood transfusion since last visit?	2 (2, 3)	65 (59, 70)	<6	—	—	—
Currently using folic acid?	99 (99, 99)	99 (98, 100)	100 (99, 100)	99 (99, 100)	35.0	0.22
Penicillin prophylaxis?	99 (98, 99)	6 (2, 16)	28 (25, 33)	55 (2, 99)	99.8	< 0.001
Malaria prophylaxis?	10 (4, 21)	99 (97, 99)	<2	—	—	—

At the consortium level, the median age at initiation of HU was 8.48 (95% CI [6.46, 10.89]) years, and the median age at enrolment was 10.14 (95% CI [7.57, 13.24]) years. Missing data for blood groups was the highest of all the variables included, with a high of 99.7% of the patients missing this data in the Ghana study population.

The prevalence of HbSS was 97% (95% CI [62%, 100%]) overall, 67% (95% CI [65%, 69%]) in Ghana, 99% (95% CI [98%, 99%]) in Nigeria, and 100% (95% CI [96%, 100%]) in Tanzania. HbSC was most common in the Ghanaian study population, with a prevalence of 33% (95% CI [31%, 34%]). The HbS/βThal genotype prevalence was consistently below 1% for all countries; however, a facility from the northwest part of Tanzania with 12 participants (see Figure S1), had an HbS/βThal prevalence of 46% (95% CI [22%, 72%]).

### 3.3 Levels of Adoption of Recommended Management Interventions

The proportion of current HU use was 12% (95% CI [5%, 24%]) overall and ranged from 6% in Tanzania to 21% in Ghana, with substantial within‐country variability (see Table 1). Blood transfusion histories differed substantially, both across and within countries. At the consortium level, 10% (95% CI [1%, 64%]) of participants had had a blood transfusion at least once; with the highest proportion found in Nigeria at 65% (95% CI [59%, 70%]) and the lowest in Ghana at 2% (95% CI [2%, 3%]). In Tanzania, 3 facilities reported no participants with a transfusion history, leading to unstable country‐level prevalence estimates. As seen in Table 1, folic acid use was high in all countries, and significant heterogeneity was also observed for penicillin prophylaxis and malaria prophylaxis.

### 3.4 Correlates of SCD Management

**FIGURE 3:**
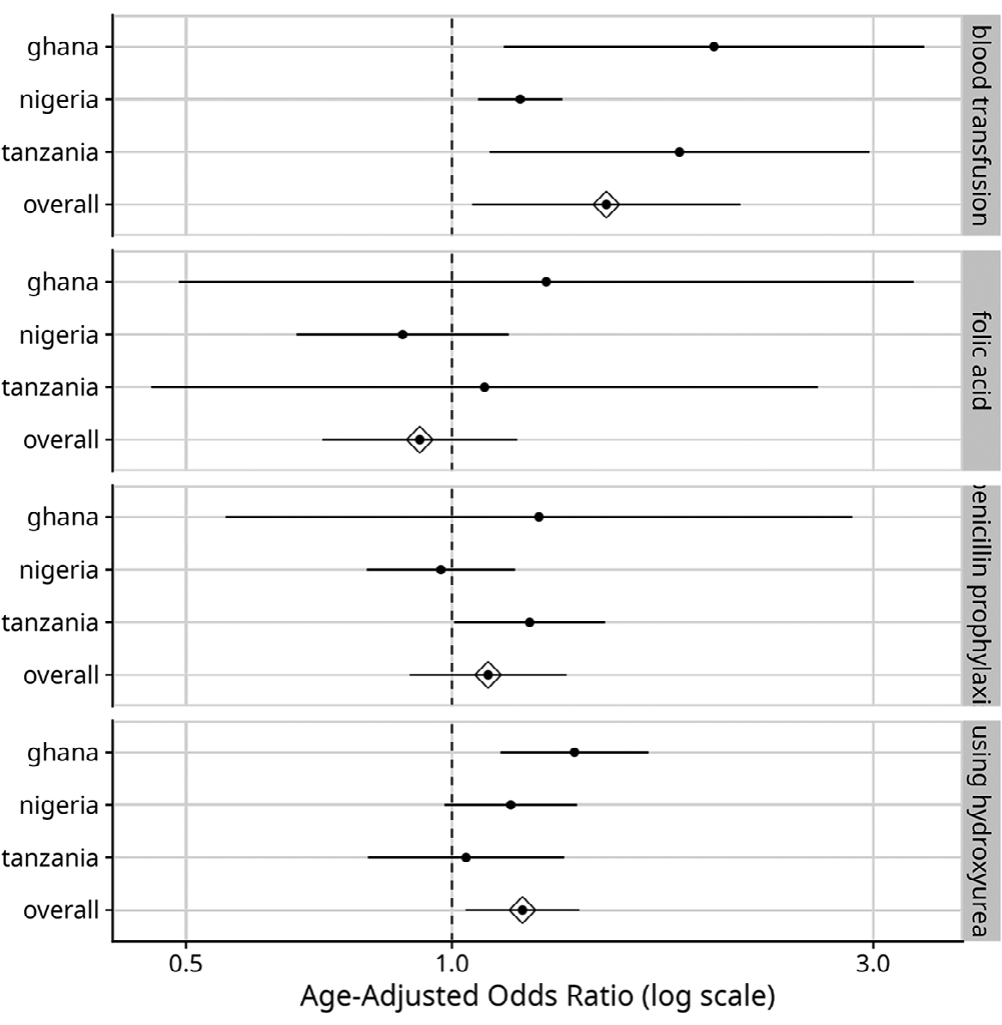
Influence of sex on SCD management. This figure presents age‐adjusted odds ratios for SCD treatment regimens among patients with the HbSS genotype (see Table S3). Females serve as the reference group, and dots represent the estimated odds ratios for males compared to females, with horizontal bars indicating 95% confidence intervals (CIs). An odds ratio greater than 1 suggests higher treatment uptake in males, while an odds ratio below 1 suggests lower uptake in males relative to females. The dashed vertical line at 1.0 represents no difference in treatment uptake between sexes.

Age and sex correlated with SCD management in HbSS participants, as shown in Table S3 and Figure 3. At the consortium level, male participants were 1.20 times more likely to be using HU than a female of the same age, and 1.50 times more likely to have had at least one blood transfusion. Males in Ghana are 1.38 times more likely to use HU than females, and 1.98 times more likely to have had at least one blood transfusion. Similarly, males in Tanzania are 1.81 times more likely to have had at least one blood transfusion.

**FIGURE 4:**
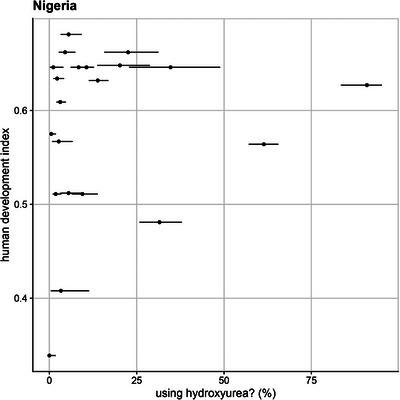
Prevalence of hydroxyurea (HU) use for each healthcare facility in Nigeria, with 95% confidence intervals (CIs). On the Y axis is the Human Development Index (HDI) of the state that a given facility is located in. The facility at the top of the plot is in a state with the highest HDI, and the facility at the bottom is in a state with the lowest HDI.

In Nigeria, since data was collected from multiple facilities across the country, the percentage of participants currently using HU varied substantially across the states and geopolitical zones. The highest uptake was observed in Edo state at 90.9% and the lowest in Kebbi state at 0%. To understand these differences, we examined whether HU use was influenced by the social and economic development of the state where each facility is located. Human Development Index (HDI) was marginally associated with HU use (*p* = 0.07; Figure 4). The full distribution of HU uptake across regions is shown in Figure S2. The HDI values for each facility are detailed in Table S4.

### 3.5 Age at HU Initiation Correlates With Age at Enrolment, Not Age at Diagnosis

**FIGURE 5:**
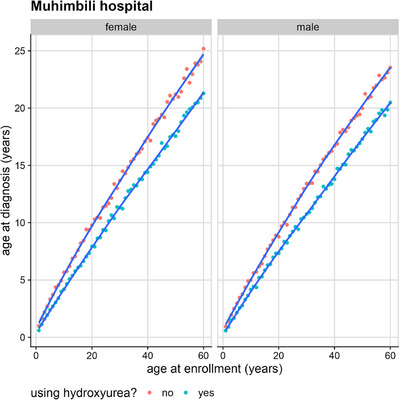
Relationship between age at hydroxyurea (HU) initiation, enrolment, and diagnosis at Muhimbili Hospital, Tanzania. This figure shows that at Muhimbili Hospital, patients on HU (blue dots and line) had a lower age at diagnosis than those not on HU (orange dots and line), suggesting that HU initiation was more closely linked to diagnosis in this setting.

Age at HU initiation was correlated with the age at enrolment in the study, but not with the age at SCD diagnosis. In a hierarchical linear model with age at HU initiation as a response variable, restricted to HbSS‐positive participants, and participants on HU at enrolment, there was no evidence of an association between age at HU initiation and age at diagnosis, at the country or consortium level, once the other factors were controlled for. Within countries, there was a significant association between age at diagnosis and HU use (*p*‐value < 0.001) for Tanzania, but not for Nigeria or Ghana. The predictions of this model for Muhimbili Hospital, in Tanzania, are illustrated in Figure 5.

### 3.6 Survival Outliers

This analysis was performed for each facility within the participating countries, with a primary focus of identifying outliers representing potential long‐term survivors, and determining factors associated with this status (Table 2).

**TABLE 2:** Summary statistics for survival outliers.

	Ghana			Nigeria			Tanzania		
Variable	Outliers	Nonoutliers	Adjusted *p*‐value	Outliers	Nonoutliers	Adjusted *p*‐value	Outliers	Nonoutliers	Adjusted *p*‐value
Sex (male/female) (%)	66.67 (12.53–98.23)	53 (51–55)	1.00	26.32 (10.12–51.42)	48.86 (47.64–50.07)	1.00	27.27 (7.33–60.68)	51.03 (49.40–52.66)	1.00
**Age (in years), median**									
at enrolment	28.67 (23.00–40.00)	10.89 (10.62–11.15)	<0.001	45.47 (40.76–50.19)	16.41 (16.14–16.68)	<0.001	48.82 (42.90–54.73)	10.94 (10.66–11.21)	<0.001
at diagnosis	13 (10.52–15.48)	1.80 (1.68–1.93)	<0.001	28.47 (22.05–34.90)	3.51 (3.39–3.63)	<0.001	36.91 (29.39–44.43)	5.46 (5.26–5.65)	<0.001
at hydroxyurea initiation	21.67 (14.50–28.84)	9.43 (9.21–9.66)	<0.05	42.05 (38.66–45.45)	13.94 (13.69–14.19)	<0.001	43.18 (36.19–50.17)	9.76 (9.51–10.02)	<0.001
**% Patients with SCD genotype**									
HbSS	66.67 (12.53–98.23)	67.21 (65.53–68.86)	1.00	73.68 (48.58–89.88)	97.27 (96.84–97.64)	<0.001	100 (67.86–100)	98.83 (98.41–99.14)	1.00
HbSC	33 (1.77–87.47)	33 (31–34)	1.00	26.32 (10.12–51.42)	2.61 (2.24–3.03)	<0.001	0 (0–32.14)	0.08 (0.02–0.26)	1.00
SBThal	—	—	—	0 (0–20.92)	0.11 (0.05–0.23)	<0.001	0 (0–32.14)	1.09 (0.79–1.50)	1.00
**% Patients using SCD treatment regimens**									
Currently, using hydroxyurea?	33.33 (1.77–87.47)	20.73 (19.32–22.21)	1.00	15.79 (4.17–40.49)	13.10 (12.30–13.95)	1.00	27.27 (7.33–60.68)	10.43 (9.47–11.48)	1.00
History of blood transfusion?	0 (0–69)	2.22 (1.75–2.82)	1.00	78.95 (53.90–93.03)	65.55 (64.38–66.70)	1.00	0 (0–32.14)	2.05 (1.62–2.57)	1.00
Currently using folic acid?	1.00 (0.31–1.00)	99.07 (98.64–99.36)	1.00	100 (79.08–100)	96.47 (95.99–96.90)	1.00	100 (67.86–100)	99.46 (99.14–99.66)	1.00
Penicillin prophylaxis?	1.00 (0.31–1.00)	0.99 (0.98–0.99)	1.00	0 (0–20.92)	11.78 (11.02–12.59)	1.00	0 (0–32.14)	29.64 (28.17–31.15)	0.92
Malaria prophylaxis?	0 (0–69)	14.66 (13.41–16.00)	1.00	100 (79.08–100)	95.37 (94.83–95.86)	1.00	0 (0–32.14)	1.31 (0.98–1.74)	1.00

We found a total of 33 outlier individuals: 3 from Ghana, 19 from Nigeria and 11 from Tanzania. There was a significant difference in SCD genotypes between the outliers and nonoutlier groups, and, as expected, a significant difference for age‐at‐enrolment (<0.001), ‐diagnosis (<0.001) and ‐hydroxyurea‐initiation (<0.001) between the outliers and nonoutlier groups in all 3 country populations. Among the outliers, the HbSS genotype was less prevalent in Nigeria (76%) compared to the nonoutliers (97%).

## 4 Discussion

This study is the first to report on a multinational SCD registry of this scale in SSA, revealing substantial differences in patient characteristics and SCD management both between and within participating countries. At the time of the study, comprehensive newborn screening programs were partially established in Ghana and Nigeria, with many registry participants recruited through these programs [14]. Although the impact of these programs on survival and quality of life remains unclear, the age at SCD diagnosis in Ghana and Nigeria was relatively low, with Ghana reporting the lowest estimates among the three countries. Ghana also had the highest proportion of the HbSC genotype, consistent with previous studies [15]. While it could be inferred that many diagnoses in Ghana are driven by programs like newborn screening rather than acute inpatient care, contrary to this, recent evidence indicates that both newborn screening and inpatient care play significant roles in SCD diagnosis in Ghana [9].

In Ghana, 33% of SCD patients have the HbSC genotype, which may partly explain lower transfusion rates [16]. Alternatively, this could underscore challenges related to the affordability and availability of blood transfusion treatment in both Ghana and Tanzania [17].

Although HU adoption levels in Ghana and Nigeria are higher than in Tanzania, this difference cannot be solely attributed to disease severity, as our study did not directly assess SCD severity across countries. Despite overall low HU adoption in all three countries, there is considerable inter‐facility variability in Nigeria, where facilities in higher‐HDI regions tended to have greater HU adoption; however, this association was only marginally significant (*p* = 0.07). This suggests that while economic and access factors may influence HU use, variability across facilities is likely driven by multiple factors beyond HDI [15]. Future studies incorporating clinical severity markers are needed to determine whether HU use differences align with disease burden across settings, and patients’ genotypes, particularly exploring possible disparity in providing HU to patients with SCD‐HbSC type, because of evolving data are suggesting that these patients do not necessarily present with milder phenotypes [18, 19].

Our finding that age at HU initiation correlates with age at enrolment, but not age at SCD diagnosis, highlights the need for increasing clinicians’ awareness for earlier intervention and more consistent treatment protocols after diagnosis. Addressing these gaps could significantly improve health outcomes for SCD patients, particularly in resource‐limited settings where early and sustained access to therapies like HU is critical. Notably, the correlation between age at HU initiation and age at enrolment seems a mere reflection of when HU usage became increasingly highlighted in medical literature as a favorable treatment option [15, 16, 17], followed by the period when enrolment was performed.

The significant association between age at diagnosis and HU use in Tanzania highlights the importance of early intervention in managing SCD within this population [15]. Our analysis reveals a consistent trend for the Muhimbili population: participants diagnosed at a younger median age are more likely to use HU, with a gap in diagnostic age ranging from one to four years across both sexes and various Tanzanian subpopulations. This finding suggests that in Tanzania, HU use may be more prevalent among patients who are diagnosed earlier, potentially reflecting a more proactive approach to managing more severe cases or those identified at a younger age. The widening of this gap with increasing age suggests that early diagnosis is crucial for HU administration, especially as patients age and encounter more clinical complications that necessitate more aggressive treatment. Despite this, overall HU use remains low across all study populations. The low HU usage in Tanzania is likely due to limited affordability, while higher usage in Ghana and Nigeria may be influenced by factors such as public‐private partnerships, local HU production, Ghana's national health insurance coverage, and the Novartis Access program [10, 11, 13, 15]. Additionally, significant variation in the adoption of other essential therapies, such as penicillin prophylaxis, was observed across and within countries, reflecting differences in healthcare policies and resources. In light of these findings, early detection and intervention in managing SCD in resource‐limited settings could improve up‐take of these therapies.

Sex was identified as a significant factor at the consortium level, with males being more likely to receive treatments such as HU and blood transfusions. This may reflect a greater burden of SCD‐related complications among males, as noted in previous studies [20, 21]. Lower fetal hemoglobin (HbF) levels, associated with greater disease severity and reduced HU response, may further contribute to this disparity [21]. However, despite these differences, we found no evidence to suggest that males are diagnosed earlier or later than females.

We show a significant difference between outlier and nonoutlier groups, where outliers were determined based on the combination of age‐at‐enrolment, ‐diagnosis, and ‐HU‐initiation. The presence of outliers, particularly those with genotypes that typically produce a more severe phenotype, such as HbSS, suggests that factors beyond genotype alone influence disease progression and treatment outcomes. The presence of these individuals in the registry is crucial, as they may reveal unique features associated with long‐term survivorship.

These findings highlight the potential of the SickleInAfrica registry to inform SCD management in SSA. Ghana, with more established newborn screening programs than Nigeria and Tanzania, had earlier average SCD diagnoses and a higher proportion of potentially less severe genotypes, that is, SC type. Hydroxyurea (HU) use was lowest in Tanzania, where it appears reserved for severe cases, but remains suboptimal across all countries. Males were more likely than females to use HU or receive blood transfusions, potentially reflecting a more severe clinical presentation or prioritization of treatment for males. The findings demonstrate the potential of the SickleInAfrica registry for advancing SCD research in SSA. Expanding facilities, increasing newborn screening coverage, improving data collection, incorporating prenatal counselling and diagnosis data reproductive decision‐making, and patient perceptions of genetic risk into future registry expansions which would provide a more comprehensive perspective on prevention strategies in SCD. Moreover, future studies should be developing advanced analytical tools are critical next steps to support research and enhance SCD management in resource‐limited settings.

## Author Contributions

RZ, GM, MJ, VN, and AW conceptualized the initial registry design and harmonized the data elements. JM, WM, AN, MJ, VN, AN, and AW created the first draft of the manuscript. JM and WM did data imputation and data analysis. JH extensively reviewed and edited the manuscript. All authors contributed to the writing and reviewing of the manuscript.

## Ethics Statement

The studies involving human participants were reviewed and approved by the Committee on Human Research Publications and Ethics (Ghana) reference CHRPE/AP/088/023, the National Health Research and Ethics Committee (Nigeria) reference NHREC/01/01/2007, by the Muhimbili University of Health and Allied Sciences and the Research and Publication Ethical Committee and the National Health Research Ethics Committee (Tanzania) reference HS2012ES, and the Human Research Ethics Committee (University of Cape Town, South Africa) reference HR015/2018. Data analysis was performed on de‐identified data.

## Patient Consent Statement

Written informed consent was obtained from all participants (or their legal guardians) before inclusion in the study. The study was approved by the institutional ethics review board as referenced above. No identifiable patient information is included in this manuscript.

## Conflicts of Interest

The authors have no competing interests.

## Supporting information



Supporting Information

Supporting Information

Supporting Information

Supporting Information

Supporting Information

Supporting Information

Supporting Information

## Data Availability

The datasets generated during and/or analyzed during the current study are available from the corresponding author upon reasonable request.

## References

[1] G. K. Mazandu , J. Hotchkiss , V. Nembaware , A. Wonkam , and N. Mulder , “The Sickle Cell Disease Ontology: Recent Development and Expansion of the Universal Sickle Cell Knowledge Representation,” Database J Biol Databases Curation 2022 (2022): baac014. 10.1093/database/baac014 PMC9216550 35363306

[2] Sickle Cell Disease Ontology Working Group. The Sickle Cell Disease Ontology: Enabling Universal Sickle Cell‐based Knowledge Representation. Database J Biol Databases Curation 2019;2019:baz118. 10.1093/database/baz118 PMC6878945 31769834

[3] B. P. D. Inusa , L. L. Hsu , N. Kohli , et al., “Sickle Cell Disease—Genetics, Pathophysiology, Clinical Presentation and Treatment,” International Journal of Neonatal Screening 5, no. 2 (2019): 20, https://www.ncbi.nlm.nih.gov/pmc/articles/PMC7510211/. —PMC [Internet]. [cited 2024 Jun 10]. Available from. 33072979 10.3390/ijns5020020 PMC7510211

[4] A. Geva , J. J. Clark , Y. Zhang , A. Popowicz , J. M. Manning , and E. J. Neufeld , “Hemoglobin Jamaica Plain—A Sickling Hemoglobin with Reduced Oxygen Affinity,” New England Journal of Medicine 351, no. 15 (2004 Oct 7): 1532–1538. 15470216 10.1056/NEJMoa040771

[5] H. F. Bunn , “Pathogenesis and Treatment of Sickle Cell Disease [Internet],” The New England Journal of Medicine 337, no. 11 (1997): 762–769, https://www.nejm.org/doi/pdf/10.1056/NEJM199709113371107. [cited 2024 Jun 10]. Available from. 9287233 10.1056/NEJM199709113371107

[6] J. Kanter and R. Kruse‐Jarres , “Management of Sickle Cell Disease From Childhood through Adulthood,” Blood Reviews 27, no. 6 (2013): 279–287. 24094945 10.1016/j.blre.2013.09.001

[7] A. B. Snyder , S. Lakshmanan , M. M. Hulihan , et al., “Surveillance for Sickle Cell Disease—Sickle Cell Data Collection Program, Two States, 2004–2018,” Mmwr Surveillance Summaries 71, no. 9 (2022): 1–18, https://www.cdc.gov/mmwr/volumes/71/ss/ss7109a1.htm. [Internet]. 2022 [cited 2024 Jun 10];71. 10.15585/mmwr.ss7109a1 PMC9552568 36201430

[8] O. Borecka , A. Ofori , F. Amini , and S. Llewellyn , “Sickle Cell Disease: A Global Patient Registry Review,” Future Rare Diseases 2 (2022 Nov 22).

[9] V. Paintsil , E. X. Amuzu , I. Nyanor , et al., “Establishing a Sickle Cell Disease Registry in Africa: Experience From the Sickle Pan‐African Research Consortium, Kumasi‐Ghana,” Frontiers in Genetics 13 (2022): 802355. 35281803 10.3389/fgene.2022.802355 PMC8908904

[10] D. Kandonga , R. Z. Sangeda , U. Masamu , et al., “Development of the Sickle Pan‐African Research Consortium Registry in Tanzania: Opportunity to Harness Data Science for Sickle Cell Disease,” Frontiers in Hematology 2 (2023): 1040720, https://www.frontiersin.org/journals/hematology/articles/10.3389/frhem.2023.1040720/full. [Internet]. [cited 2024 Jun 10];2. Available from. 39247216 10.3389/frhem.2023.1040720 PMC11378979

[11] N. S. Munung , V. Nembaware , J. de Vries , et al., “Establishing a Multi‐Country Sickle Cell Disease Registry in Africa: Ethical Considerations,” Frontiers in Genetics 10 (2019): 943. 31649726 10.3389/fgene.2019.00943 PMC6795756

[12] L. H. Pecker , B. A. Schaefer , and L. Luchtman‐Jones , “Knowledge Insufficient: The Management of Haemoglobin SC Disease,” British Journal of Haematology 176, no. 4 (2017): 515–526. 27982424 10.1111/bjh.14444 PMC5303157

[13] C. Elendu , D. C. Amaechi , C. E. Alakwe‐Ojimba , et al., “Understanding Sickle Cell Disease: Causes, Symptoms, and Treatment Options,” Medicine 102, no. 38 (2023): e35237. 37746969 10.1097/MD.0000000000035237 PMC10519513

[14] B. L. Therrell , M. A. Lloyd‐Puryear , K. Ohene‐Frempong , et al., “Empowering Newborn Screening Programs in African Countries Through Establishment of an International Collaborative Effort,” Journal of Community Genetics 11, no. 3 (2020): 253–268. 32415570 10.1007/s12687-020-00463-7 PMC7295888

[15] Burden of Sickle Cell Disease in Ghana : The Korle‐Bu Experience—Asare—2018—Advances in Hematology—Wiley Online Library [Internet]. [cited 2024 Aug 21], https://onlinelibrary.wiley.com/doi/10.1155/2018/6161270. 10.1155/2018/6161270 PMC6304501 30631363

[16] L. A. Boateng , A. D. Campbell , R. D. Davenport , et al., “Red Blood Cell Alloimmunization and Minor Red Blood Cell Antigen Phenotypes in Transfused Ghanaian Patients With Sickle Cell Disease,” Transfusion 59, no. 6 (2019): 2016–2022. 30758856 10.1111/trf.15197

[17] G. N. Doku , W. K. Agbozo , R. A. Annor , P. E. Mawudzro , and E. E. Agbeli , “Frequencies and Ethnic Distribution of ABO and RhD Blood Groups in the Volta Region of Ghana, Towards Effective Blood Bank Services,” African Health Sciences 22, no. 1 (2022): 641–647. 10.4314/ahs.v22i1.74 PMC9382516 36032446

[18] W. K. Ghunney , E. V. Asare , J. B. Ayete‐Nyampong , et al., “Most Adults With Severe HbSC Disease Are Not Treated With Hydroxyurea,” Blood Advances 7, no. 13 (2023): 3312–3319. 36799926 10.1182/bloodadvances.2022009049 PMC10362538

[19] M. Nelson , L. Noisette , N. Pugh , et al., “The Clinical Spectrum of HbSC Sickle Cell Disease—Not a Benign Condition,” British Journal of Haematology 205, no. 2 (2024): 653–663. 38898714 10.1111/bjh.19523 PMC11315634

[20] S. Lanzkron , J.r Haywood C , and P. Fagan , “Documenting the Effectiveness of Hydroxyurea (HU) to Treat Sickle Cell Disease (SCD) in the Community Setting,” Blood 110, no. 11 (2007): 956, https://onlinelibrary.wiley.com/doi/pdfdirect/10.1002/ajh.27074. Available from.

[21] M. Di Mauro , S. El Hoss , A. Nardo‐Marino , et al., “Males With Sickle Cell Disease Have Higher Risks of Cerebrovascular Disease, Increased Inflammation, and a Reduced Response to Hydroxyurea,” American Journal of Hematology 98 (2023): E341–E344. 37646569 10.1002/ajh.27074

