# Supplementary material for: Sickle Cell Disease in Africa: SickleInAfrica Registry in Ghana, Nigeria and Tanzania
Source: EJHaem. 2025 May 6;6(3):e70044. doi: 10.1002/jha2.70044 (PMC12053511; doi:10.1002/jha2.70044)
Supplement: Supplementary file 1 — Supporting Information [file JHA2-6-e70044-s001.docx]

**Supplementary Table S1: Complete List of Variables Collected in the Registry.** This table provides a comprehensive list of all 92 variables collected in the registry, including those analyzed in the study and additional variables that were recorded but not included in the primary analysis.

|  | **Core data elements** | **Field attribute** |
| --- | --- | --- |
| **Consent** | | |
| 1 | SickleInAfrica participant number | NA |
| 2 | Has the patient consented? | NA |
| 3 | Type(s) of informed consent obtained | NA |
| 4 | Date subject signed consent | NA |
| 5 | Consented by: | NA |
| **Demographics** | | |
| 6 | Visit date | NA |
| 7 | Type of visit | NA |
| 8 | Site participant number | NA |
| 9 | Medical record number | NA |
| 10 | Hospital name | NA |
| 11 | First name | NA |
| 12 | Middle name | NA |
| 13 | Last name | NA |
| 14 | Date of birth | Date of birth |
| 15 | Calculated age (in months) | Age |
| 16 | Self-reported age | Age |
| 17 | Height/Length | Height (centimeter) |
| 18 | Weight | Weight (kilogram) |
| 19 | Respondent’s sex | Sex |
| 20 | Current marital status | What is your current marital status? |
| 21 | Ethnic group | NA |
| 22 | Religion | NA |
| 23 | Region | NA |
| 24 | Street | NA |
| 25 | Nearest landmark | NA |
| 26 | Telephone 1 | NA |
| 27 | Telephone 2 | NA |
| 28 | Next of kin name | NA |
| 29 | Next of kin telephone number | NA |
| 30 | Relationship to participant | NA |
| **SCD Diagnosis Details** | | |
| 31 | Is the date of SCD diagnosis known? | NA |
| 32 | Date of SCD diagnosis | NA |
| 33 | SCD test result | NA |
| 34 | If other SCD test result, please specify | NA |
| 35 | If other test, please specify | NA |
| 36 | ABO blood group | NA |
| 37 | Type of test? | NA |
| **Management Details** | | |
| 38 | Using hydroxyurea | NA |
| 39 | Date of initiation of hydroxyurea therapy | NA |
| 40 | Using penicillin V (prophylaxis) | NA |
| 41 | Using malaria chemoprophylaxis | NA |
| 42 | Using folic acid | NA |
| 43 | Pneumococcal vaccination up to date | NA |
| 44 | Blood transfusion | NA |
| 45 | Date of blood transfusion | NA |
| 46 | Units transfused | NA |
| **Vital Signs** | | |
| 47 | Patient’s body temperature | Temperature (Celsius) |
| 48 | Type of body temperature taken | Type of body temperature taken |
| 49 | Patient’s respiratory rate | Patient’s respiratory rate |
| 50 | Difficulty in breathing | NA |
| 51 | Systolic blood pressure | Blood pressure systolic |
| 52 | Diastolic blood pressure | Blood pressure diastolic |
| 53 | Priapism | NA |
| 54 | Chest pain | NA |
| 55 | Anemia | NA |
| 56 | Jaundice | NA |
| 57 | If jaundice is present, please provide additional information | NA |
| **Kidney Function Assay** | | |
| 58 | Serum creatinine concentration | NA |
| 59 | Urea | NA |
| 60 | Urinary albumin concentration | NA |
| 61 | Urinary creatinine concentration | NA |
| **Liver Function Assay** | | |
| 62 | Alanine aminotransferase level | NA |
| 63 | Aspartate aminotransferase level | NA |
| 64 | Alkaline phosphatase level | NA |
| **Laboratory Results: Complete Blood Count** | | |
| 65 | Red blood cell count (RBC) | Red cell count  (million cells/uL) |
| 66 | White blood cell count (WBC) | White blood cell count (1000 cells/uL) |
| 67 | Platelet count | Platelet count  (1000 cells/uL) |
| 68 | Hemoglobin | Hemoglobin (g/dL) |
| 69 | Mean cell volume (MCV) | Mean cell volume (fL) |
| 70 | Mean cell hemoglobin (MCH) | Mean cell hemoglobin (pg) |
| 71 | Mean cell hemoglobin concentration (MCHC) | MCHC (g/dL) |
| 72 | Red cell distribution width (RDW) | Red cell distribution width (%) |
| **Laboratory Results: Bilirubin Level** | | |
| 73 | Total bilirubin concentration | NA |
| **Laboratory Results: Lactate Dehydrogenase Level** | | |
| 74 | Lactate dehydrogenase level | NA |
| **Laboratory Results: Reticulocyte Level** | | |
| 75 | Number of reticulocytes | NA |
| **Laboratory Results: Hemoglobin Characterisation** | | |
| 76 | For which hemoglobins were assay results recorded? | NA |
| 77 | Record the levels Hb A, if measured | NA |
| 78 | Record the levels Hb F, if measured | NA |
| 79 | Record the levels Hb S, if measured | NA |
| 80 | Record the levels Hb C, if measured | NA |
| 81 | Record the levels Hb E, if measured | NA |
| 82 | Record the levels Hb A2, if measured | NA |
| 83 | Record the levels Hb D-Punjab, if measured | NA |
| 84 | Record the levels Hb G-Philadelphia, if measured | NA |
| 85 | Record the levels Hb O-Arab, if measured | NA |
| 86 | Total hemoglobin (Hbtotal) | NA |
| **Arterial blood gas - ABG** | | |
| 87 | Partial pressure of carbon dioxide (PaCO2) | NA |
| 88 | Partial pressure of oxygen (PaO2) | NA |
| 89 | Oxyhemoglobin saturation (HbO2) | NA |
| 90 | Carboxyhemoglobin (COHb) | NA |
| 91 | Methemoglobin (MetHb) | NA |
| 92 | Complete? | NA |
