# Supplementary material for: Sickle Cell Disease in Africa: SickleInAfrica Registry in Ghana, Nigeria and Tanzania
Source: EJHaem. 2025 May 6;6(3):e70044. doi: 10.1002/jha2.70044 (PMC12053511; doi:10.1002/jha2.70044)
Supplement: Supplementary file 2 — Supporting Information [file JHA2-6-e70044-s002.docx]

**Supplementary Table S2:** SickleInAfrica Phase 1 recruitment facilities across Ghana, Nigeria, and Tanzania, by country, hospital name, geographical location, and patient records.

| **Country** | **Hospital Name** | **Latitude (^o^)** | **Longitude (^o^)** | **Records** |
| --- | --- | --- | --- | --- |
| Nigeria | University Of Abuja Teaching Hospital | 8.95 | 7.06 | 918 |
| Nigeria | University Of Nigeria Teaching Hospital | 7.15 | 7.80 | 406 |
| Nigeria | University College Hospital | 7.40 | 3.90 | 585 |
| Nigeria | Zankli Medical Centre | 9.08 | 7.45 | 49 |
| Nigeria | General Hospital Nyanya | 9.03 | 7.57 | 178 |
| Nigeria | Federal Medical Centre Keffi | 8.85 | 7.89 | 378 |
| Nigeria | Nnamdi Azikiwe University Teaching Hospital | 6.02 | 6.91 | 312 |
| Nigeria | Federal Medical Centre, Birnin Kebbi | 12.45 | 4.20 | 202 |
| Nigeria | University of Maidugiri Teaching Hospital | 11.83 | 13.18 | 200 |
| Nigeria | Maitama General Hospital | 9.09 | 7.48 | 44 |
| Nigeria | Ahmadu Bello University Teaching Hospital | 10.53 | 7.43 | 499 |
| Nigeria | National Hospital Abuja | 9.04 | 7.46 | 418 |
| Nigeria | Federal Teaching Hospital Abakaliki | 6.33 | 8.11 | 150 |
| Nigeria | Irrua Specialist Teaching Hospital | 6.73 | 6.19 | 99 |
| Nigeria | Lagos University Teaching Hospital | 6.52 | 3.35 | 236 |
| Nigeria | Aminu Kano Teaching Hospital | 11.97 | 8.55 | 222 |
| Nigeria | Barau Dikko Hospital Kaduna | 10.53 | 7.44 | 243 |
| Nigeria | Obafemi Awolowo University Teaching Hospital | 7.49 | 4.55 | 647 |
| Nigeria | Jos University Teaching Hospital | 9.92 | 8.89 | 505 |
| Nigeria | Rivers State University Teaching Hospital | 4.78 | 7.01 | 109 |
| Nigeria | Federal Teaching Hospital Gombe | 10.30 | 11.14 | 61 |
| Nigeria | Federal Medical Centre Asaba | 6.20 | 6.74 | - |
| Tanzania | Bugando Medical Center | -2.53 | 32.91 | 651 |
| Tanzania | Temeke Regional Referral Hospital | -6.86 | 39.26 | 994 |
| Tanzania | Amana Regional Referral Hospital | -6.83 | 39.26 | 795 |
| Tanzania | Muhimbili Mloganzila | -6.82 | 39.06 | 104 |
| Tanzania | Mwananyamala Regional Referral Hospital | -6.79 | 39.25 | 651 |
| Tanzania | Bagamoyo District Hospital | -6.44 | 38.91 | 131 |
| Tanzania | Shree Hindu Mandal | -6.67 | 39.21 | 13 |
| Tanzania | Muhimbili National Hospital | -6.80 | 39.27 | 321 |
| Ghana | Komfo Anokye Teaching Hospital | 6.70 | -1.63 | 3146 |
