# Supplementary material for: Sickle Cell Disease in Africa: SickleInAfrica Registry in Ghana, Nigeria and Tanzania
Source: EJHaem. 2025 May 6;6(3):e70044. doi: 10.1002/jha2.70044 (PMC12053511; doi:10.1002/jha2.70044)
Supplement: Supplementary file 3 — Supporting Information [file JHA2-6-e70044-s005.docx]

**Supplementary Table S3**. **Influence of sex on SCD management.** Showing age-adjusted odds ratios for responses to registry questions relating to SCD management, with sex as the predictor, for the subset of patients in the registry with the HbSS genotype.

| **Response** | **Age-adjusted odds ratio (95% CI)** | | | | **p-value** |
| --- | --- | --- | --- | --- | --- |
|  | **Ghana** | **Nigeria** | **Tanzania** | **Overall** |  |
| **Currently using hydroxyurea?** | 1.38  (1.14, 1.67) | 1.17  (0.98, 1.39) | 1.04  (0.80, 1.34) | 1.20  (1.04, 1.39) | 0.01 |
| **Blood transfusion since last visit?** | 1.98  (1.15, 3.43) | 1.19  (1.07, 1.33) | 1.81  (1.10, 3.00) | 1.50  (1.05, 2.12) | 0.02 |
| **Currently using folic acid?** | 1.28  (0.49, 3.34) | 0.88  (0.67, 1.16) | 1.09  (0.46, 2.59) | 0.92  (0.71, 1.19) | 0.52 |
| **Penicillin prophylaxis?** | 1.21  (0.53, 2.74) | 0.97  (0.80, 1.18) | 1.22  (1.00, 1.48) | 1.09  (0.90, 1.34) | 0.38 |
