# Supplementary material for: Sickle Cell Disease in Africa: SickleInAfrica Registry in Ghana, Nigeria and Tanzania
Source: EJHaem. 2025 May 6;6(3):e70044. doi: 10.1002/jha2.70044 (PMC12053511; doi:10.1002/jha2.70044)
Supplement: Supplementary file 4 — Supporting Information [file JHA2-6-e70044-s004.docx]

**Supplementary Table S4:** Recruitment facilities in Nigeria illustrated by hospital name, state, patient records, and state Human Development Index (HDI).

| **Hospital name** | **State** | **Records** | **State HDI*** |
| --- | --- | --- | --- |
| Ahmadu Bello University Teaching Hospital | Kaduna | 499 | 0.511 |
| Aminu Kano Teaching Hospital | Kano | 222 | 0.481 |
| Barau Dikko Hospital Kaduna | Kaduna | 243 | 0.511 |
| Federal Medical Centre Asaba | Delta | 111 | 0.662 |
| Federal Medical Centre Keffi | Nasarawa | 378 | 0.575 |
| Federal Medical Centre, Birnin Kebbi | Kebbi | 202 | 0.339 |
| Federal Teaching Hospital Abakaliki | Ebonyi | 150 | 0.567 |
| Federal Teaching Hospital Gombe | Gombe | 61 | 0.408 |
| General Hospital Nyanya | FCT | 178 | 0.646 |
| Irrua Specialist Teaching Hospital | Edo | 99 | 0.627 |
| Jos University Teaching Hospital | Plateau | 505 | 0.564 |
| Lagos University Teaching Hospital | Lagos | 236 | 0.681 |
| Maitama General Hospital | FCT | 44 | 0.646 |
| National Hospital Abuja | FCT | 418 | 0.646 |
| Nnamdi Azikiwe University Teaching Hospital | Anambra | 312 | 0.662 |
| Obafemi Awolowo University Teaching Hospital | Osun | 647 | 0.609 |
| Rivers State University Teaching Hospital | Rivers | 109 | 0.648 |
| University College Hospital | Oyo | 585 | 0.632 |
| University Of Abuja Teaching Hospital | FCT | 918 | 0.646 |
| University Of Maidugiri Teaching Hospital | Borno | 200 | 0.512 |
| University Of Nigeria Teaching Hospital | Enugu | 406 | 0.634 |
| Zankli Medical Centre | FCT | 49 | 0.646 |
