# Supplementary figures and images for: Sickle Cell Disease in Africa: SickleInAfrica Registry in Ghana, Nigeria and Tanzania
Source: EJHaem. 2025 May 6;6(3):e70044. doi: 10.1002/jha2.70044 (PMC12053511; doi:10.1002/jha2.70044)

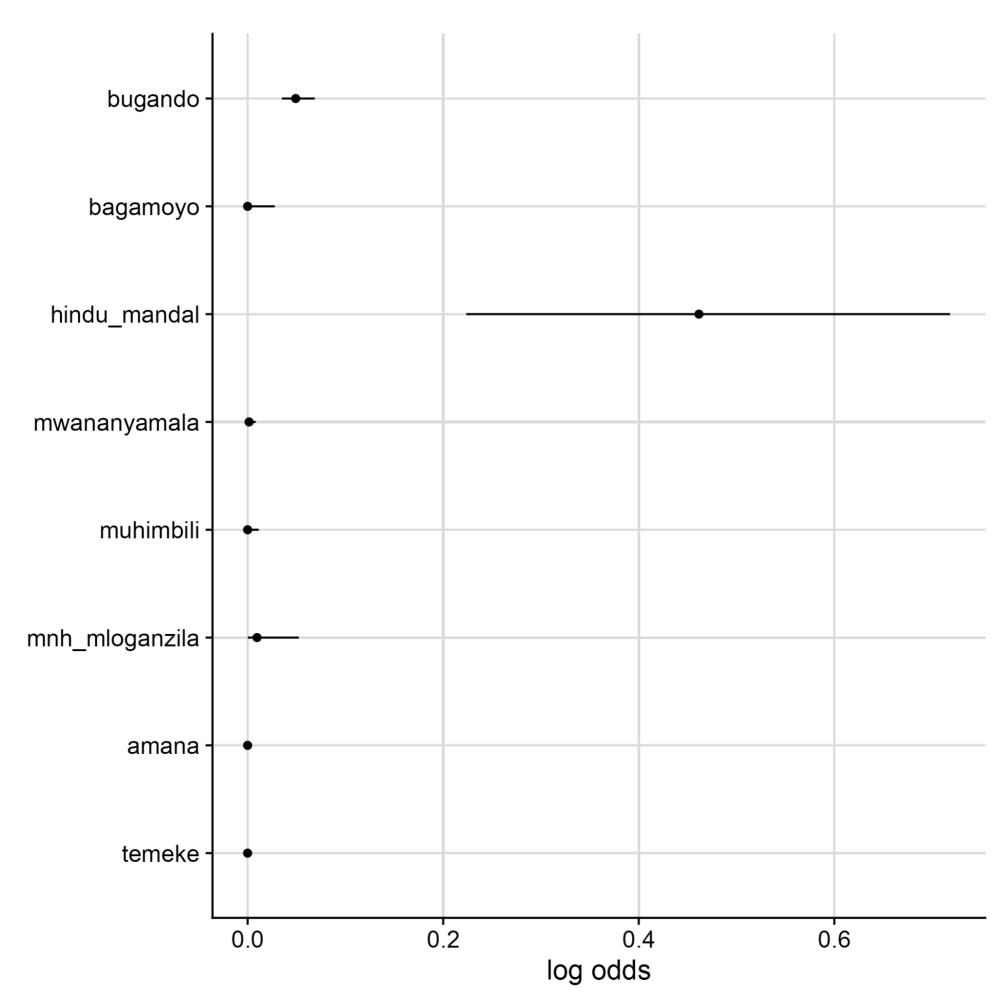

Supplement: Supplementary file 5 — Supporting Information [file JHA2-6-e70044-s007.tif]

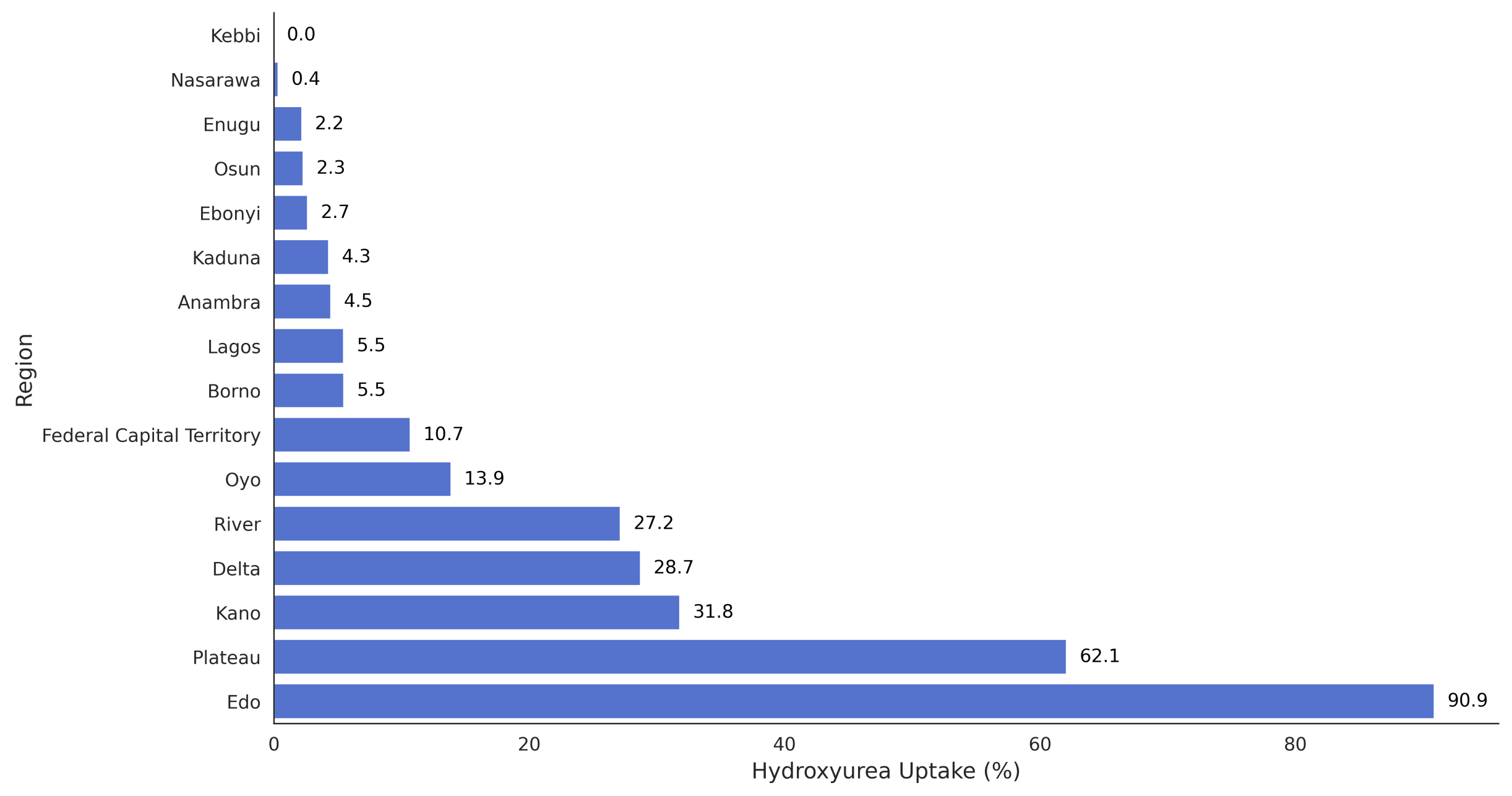

Supplement: Supplementary file 6 — Supporting Information [file JHA2-6-e70044-s003.tif]
