# Supplementary material for: Sickle Cell Disease in Africa: SickleInAfrica Registry in Ghana, Nigeria and Tanzania
Source: EJHaem. 2025 May 6;6(3):e70044. doi: 10.1002/jha2.70044 (PMC12053511; doi:10.1002/jha2.70044)
Supplement: Supplementary file 7 — Supporting Information [file JHA2-6-e70044-s006.docx]

**Supplementary Table S1: Complete List of Variables Collected in the Registry.** This table provides a comprehensive list of all 92 variables collected in the registry, including those analyzed in the study and additional variables that were recorded but not included in the primary analysis.

|  | **Core data elements** | **Field attribute** |
| --- | --- | --- |
| **Consent** | | |
| 1 | SickleInAfrica participant number | NA |
| 2 | Has the patient consented? | NA |
| 3 | Type(s) of informed consent obtained | NA |
| 4 | Date subject signed consent | NA |
| 5 | Consented by: | NA |
| **Demographics** | | |
| 6 | Visit date | NA |
| 7 | Type of visit | NA |
| 8 | Site participant number | NA |
| 9 | Medical record number | NA |
| 10 | Hospital name | NA |
| 11 | First name | NA |
| 12 | Middle name | NA |
| 13 | Last name | NA |
| 14 | Date of birth | Date of birth |
| 15 | Calculated age (in months) | Age |
| 16 | Self-reported age | Age |
| 17 | Height/Length | Height (centimeter) |
| 18 | Weight | Weight (kilogram) |
| 19 | Respondent’s sex | Sex |
| 20 | Current marital status | What is your current marital status? |
| 21 | Ethnic group | NA |
| 22 | Religion | NA |
| 23 | Region | NA |
| 24 | Street | NA |
| 25 | Nearest landmark | NA |
| 26 | Telephone 1 | NA |
| 27 | Telephone 2 | NA |
| 28 | Next of kin name | NA |
| 29 | Next of kin telephone number | NA |
| 30 | Relationship to participant | NA |
| **SCD Diagnosis Details** | | |
| 31 | Is the date of SCD diagnosis known? | NA |
| 32 | Date of SCD diagnosis | NA |
| 33 | SCD test result | NA |
| 34 | If other SCD test result, please specify | NA |
| 35 | If other test, please specify | NA |
| 36 | ABO blood group | NA |
| 37 | Type of test? | NA |
| **Management Details** | | |
| 38 | Using hydroxyurea | NA |
| 39 | Date of initiation of hydroxyurea therapy | NA |
| 40 | Using penicillin V (prophylaxis) | NA |
| 41 | Using malaria chemoprophylaxis | NA |
| 42 | Using folic acid | NA |
| 43 | Pneumococcal vaccination up to date | NA |
| 44 | Blood transfusion | NA |
| 45 | Date of blood transfusion | NA |
| 46 | Units transfused | NA |
| **Vital Signs** | | |
| 47 | Patient’s body temperature | Temperature (Celsius) |
| 48 | Type of body temperature taken | Type of body temperature taken |
| 49 | Patient’s respiratory rate | Patient’s respiratory rate |
| 50 | Difficulty in breathing | NA |
| 51 | Systolic blood pressure | Blood pressure systolic |
| 52 | Diastolic blood pressure | Blood pressure diastolic |
| 53 | Priapism | NA |
| 54 | Chest pain | NA |
| 55 | Anemia | NA |
| 56 | Jaundice | NA |
| 57 | If jaundice is present, please provide additional information | NA |
| **Kidney Function Assay** | | |
| 58 | Serum creatinine concentration | NA |
| 59 | Urea | NA |
| 60 | Urinary albumin concentration | NA |
| 61 | Urinary creatinine concentration | NA |
| **Liver Function Assay** | | |
| 62 | Alanine aminotransferase level | NA |
| 63 | Aspartate aminotransferase level | NA |
| 64 | Alkaline phosphatase level | NA |
| **Laboratory Results: Complete Blood Count** | | |
| 65 | Red blood cell count (RBC) | Red cell count  (million cells/uL) |
| 66 | White blood cell count (WBC) | White blood cell count (1000 cells/uL) |
| 67 | Platelet count | Platelet count  (1000 cells/uL) |
| 68 | Hemoglobin | Hemoglobin (g/dL) |
| 69 | Mean cell volume (MCV) | Mean cell volume (fL) |
| 70 | Mean cell hemoglobin (MCH) | Mean cell hemoglobin (pg) |
| 71 | Mean cell hemoglobin concentration (MCHC) | MCHC (g/dL) |
| 72 | Red cell distribution width (RDW) | Red cell distribution width (%) |
| **Laboratory Results: Bilirubin Level** | | |
| 73 | Total bilirubin concentration | NA |
| **Laboratory Results: Lactate Dehydrogenase Level** | | |
| 74 | Lactate dehydrogenase level | NA |
| **Laboratory Results: Reticulocyte Level** | | |
| 75 | Number of reticulocytes | NA |
| **Laboratory Results: Hemoglobin Characterisation** | | |
| 76 | For which hemoglobins were assay results recorded? | NA |
| 77 | Record the levels Hb A, if measured | NA |
| 78 | Record the levels Hb F, if measured | NA |
| 79 | Record the levels Hb S, if measured | NA |
| 80 | Record the levels Hb C, if measured | NA |
| 81 | Record the levels Hb E, if measured | NA |
| 82 | Record the levels Hb A2, if measured | NA |
| 83 | Record the levels Hb D-Punjab, if measured | NA |
| 84 | Record the levels Hb G-Philadelphia, if measured | NA |
| 85 | Record the levels Hb O-Arab, if measured | NA |
| 86 | Total hemoglobin (Hbtotal) | NA |
| **Arterial blood gas - ABG** | | |
| 87 | Partial pressure of carbon dioxide (PaCO2) | NA |
| 88 | Partial pressure of oxygen (PaO2) | NA |
| 89 | Oxyhemoglobin saturation (HbO2) | NA |
| 90 | Carboxyhemoglobin (COHb) | NA |
| 91 | Methemoglobin (MetHb) | NA |
| 92 | Complete? | NA |

**Supplementary Table S2:** SickleInAfrica Phase 1 recruitment facilities across Ghana, Nigeria, and Tanzania, by country, hospital name, geographical location, and patient records.

| **Country** | **Hospital Name** | **Latitude (^o^)** | **Longitude (^o^)** | **Records** |
| --- | --- | --- | --- | --- |
| Nigeria | University Of Abuja Teaching Hospital | 8.95 | 7.06 | 918 |
| Nigeria | University Of Nigeria Teaching Hospital | 7.15 | 7.80 | 406 |
| Nigeria | University College Hospital | 7.40 | 3.90 | 585 |
| Nigeria | Zankli Medical Centre | 9.08 | 7.45 | 49 |
| Nigeria | General Hospital Nyanya | 9.03 | 7.57 | 178 |
| Nigeria | Federal Medical Centre Keffi | 8.85 | 7.89 | 378 |
| Nigeria | Nnamdi Azikiwe University Teaching Hospital | 6.02 | 6.91 | 312 |
| Nigeria | Federal Medical Centre, Birnin Kebbi | 12.45 | 4.20 | 202 |
| Nigeria | University of Maidugiri Teaching Hospital | 11.83 | 13.18 | 200 |
| Nigeria | Maitama General Hospital | 9.09 | 7.48 | 44 |
| Nigeria | Ahmadu Bello University Teaching Hospital | 10.53 | 7.43 | 499 |
| Nigeria | National Hospital Abuja | 9.04 | 7.46 | 418 |
| Nigeria | Federal Teaching Hospital Abakaliki | 6.33 | 8.11 | 150 |
| Nigeria | Irrua Specialist Teaching Hospital | 6.73 | 6.19 | 99 |
| Nigeria | Lagos University Teaching Hospital | 6.52 | 3.35 | 236 |
| Nigeria | Aminu Kano Teaching Hospital | 11.97 | 8.55 | 222 |
| Nigeria | Barau Dikko Hospital Kaduna | 10.53 | 7.44 | 243 |
| Nigeria | Obafemi Awolowo University Teaching Hospital | 7.49 | 4.55 | 647 |
| Nigeria | Jos University Teaching Hospital | 9.92 | 8.89 | 505 |
| Nigeria | Rivers State University Teaching Hospital | 4.78 | 7.01 | 109 |
| Nigeria | Federal Teaching Hospital Gombe | 10.30 | 11.14 | 61 |
| Nigeria | Federal Medical Centre Asaba | 6.20 | 6.74 | - |
| Tanzania | Bugando Medical Center | -2.53 | 32.91 | 651 |
| Tanzania | Temeke Regional Referral Hospital | -6.86 | 39.26 | 994 |
| Tanzania | Amana Regional Referral Hospital | -6.83 | 39.26 | 795 |
| Tanzania | Muhimbili Mloganzila | -6.82 | 39.06 | 104 |
| Tanzania | Mwananyamala Regional Referral Hospital | -6.79 | 39.25 | 651 |
| Tanzania | Bagamoyo District Hospital | -6.44 | 38.91 | 131 |
| Tanzania | Shree Hindu Mandal | -6.67 | 39.21 | 13 |
| Tanzania | Muhimbili National Hospital | -6.80 | 39.27 | 321 |
| Ghana | Komfo Anokye Teaching Hospital | 6.70 | -1.63 | 3146 |

**Supplementary Table S3**. **Influence of sex on SCD management.** Showing age-adjusted odds ratios for responses to registry questions relating to SCD management, with sex as the predictor, for the subset of patients in the registry with the HbSS genotype.

| **Response** | **Age-adjusted odds ratio (95% CI)** | | | | **p-value** |
| --- | --- | --- | --- | --- | --- |
|  | **Ghana** | **Nigeria** | **Tanzania** | **Overall** |  |
| **Currently using hydroxyurea?** | 1.38  (1.14, 1.67) | 1.17  (0.98, 1.39) | 1.04  (0.80, 1.34) | 1.20  (1.04, 1.39) | 0.01 |
| **Blood transfusion since last visit?** | 1.98  (1.15, 3.43) | 1.19  (1.07, 1.33) | 1.81  (1.10, 3.00) | 1.50  (1.05, 2.12) | 0.02 |
| **Currently using folic acid?** | 1.28  (0.49, 3.34) | 0.88  (0.67, 1.16) | 1.09  (0.46, 2.59) | 0.92  (0.71, 1.19) | 0.52 |
| **Penicillin prophylaxis?** | 1.21  (0.53, 2.74) | 0.97  (0.80, 1.18) | 1.22  (1.00, 1.48) | 1.09  (0.90, 1.34) | 0.38 |

**Supplementary Table S4:** Recruitment facilities in Nigeria illustrated by hospital name, state, patient records, and state Human Development Index (HDI).

| **Hospital name** | **State** | **Records** | **State HDI*** |
| --- | --- | --- | --- |
| Ahmadu Bello University Teaching Hospital | Kaduna | 499 | 0.511 |
| Aminu Kano Teaching Hospital | Kano | 222 | 0.481 |
| Barau Dikko Hospital Kaduna | Kaduna | 243 | 0.511 |
| Federal Medical Centre Asaba | Delta | 111 | 0.662 |
| Federal Medical Centre Keffi | Nasarawa | 378 | 0.575 |
| Federal Medical Centre, Birnin Kebbi | Kebbi | 202 | 0.339 |
| Federal Teaching Hospital Abakaliki | Ebonyi | 150 | 0.567 |
| Federal Teaching Hospital Gombe | Gombe | 61 | 0.408 |
| General Hospital Nyanya | FCT | 178 | 0.646 |
| Irrua Specialist Teaching Hospital | Edo | 99 | 0.627 |
| Jos University Teaching Hospital | Plateau | 505 | 0.564 |
| Lagos University Teaching Hospital | Lagos | 236 | 0.681 |
| Maitama General Hospital | FCT | 44 | 0.646 |
| National Hospital Abuja | FCT | 418 | 0.646 |
| Nnamdi Azikiwe University Teaching Hospital | Anambra | 312 | 0.662 |
| Obafemi Awolowo University Teaching Hospital | Osun | 647 | 0.609 |
| Rivers State University Teaching Hospital | Rivers | 109 | 0.648 |
| University College Hospital | Oyo | 585 | 0.632 |
| University Of Abuja Teaching Hospital | FCT | 918 | 0.646 |
| University Of Maidugiri Teaching Hospital | Borno | 200 | 0.512 |
| University Of Nigeria Teaching Hospital | Enugu | 406 | 0.634 |
| Zankli Medical Centre | FCT | 49 | 0.646 |
